# Innate-like T cells in children with sickle cell disease

**DOI:** 10.1371/journal.pone.0219047

**Published:** 2019-06-28

**Authors:** Slimane Allali, Céline Dietrich, François Machavoine, Rachel Rignault-Bricard, Valentine Brousse, Mariane de Montalembert, Olivier Hermine, Thiago Trovati Maciel, Maria Leite-de-Moraes

**Affiliations:** 1 Department of General Pediatrics and Pediatric Infectious Diseases, Hôpital Necker-Enfants malades, Paris Descartes – Sorbonne Paris Cité University, Assistance Publique-Hôpitaux de Paris, Paris, France; 2 Laboratory of Cellular and Molecular Mechanisms of Hematological Disorders and Therapeutical Implications, Paris Descartes – Sorbonne Paris Cité University, Imagine Institute, Inserm U1163, Paris, France; 3 Laboratory of Excellence GR-Ex, Paris, France; 4 Laboratory of Immunoregulation and Immunopathology, Institut Necker-Enfants malades, Centre National de la Recherche Scientifique (CNRS) UMR 8253, Inserm UMR 1151, Paris Descartes – Sorbonne Paris Cité University, Paris, France; 5 Department of Hematology, Hôpital Necker-Enfants malades, Paris Descartes – Sorbonne Paris Cité University, Assistance Publique-Hôpitaux de Paris, Paris, France; Université Claude Bernard Lyon 1, FRANCE

## Abstract

**Background:**

The implication of lymphocytes in sickle cell disease pathogenesis is supported by a number of recent reports. These studies provided evidence for the activation of invariant natural killer T (iNKT) cells in adult patients, but did not investigate the involvement of other innate-like T cell subsets so far.

**Methods:**

Here we present a monocentric prospective observational study evaluating the number and functional properties of both circulating conventional and innate-like T cells, namely iNKT, Mucosal-Associated Invariant T (MAIT) and gammadelta (γδ) T cells in a cohort of 39 children with sickle cell disease.

**Results:**

Relative to age-matched healthy controls, we found that patients had a higher frequency of IL-13- and IL-17-producing CD4^+^ T cells, as well as higher MAIT cell counts with an increased frequency of IL-17-producing MAIT cells. Patients also presented increased Vδ2 γδ T cell counts, especially during vaso-occlusive crisis, and a lower frequency of IFNγ-producing Vδ2 γδ T cells, except during crisis. iNKT cell counts and the frequency of IFNγ-producing iNKT cells were unchanged compared to controls. Our study revealed positive correlations between 1) the frequency of IFNγ-producing CD4^+^, CD8^+^ and Vδ2 γδ T cells and the number of hospitalizations for vaso-occlusive crisis in the previous year; 2) the frequency of IFNγ-producing iNKT cells and patients’ age and 3) the frequency of IL-17-producing Vδ2 γδ T cells and hemoglobin S level.

**Conclusion:**

These results strongly suggest a role of innate-like T cells in sickle cell disease pathophysiology, especially that of IL-17-producing MAIT and γδ T cells.

## Introduction

Sickle cell disease (SCD) is a common life-threatening genetic hemoglobin disorder affecting millions of people worldwide and characterized by chronic hemolysis, recurrent painful vaso-occlusive events and progressive organ damage [1]. It originates from a single nucleotide mutation of the β-globin gene, leading to polymerization of the abnormal deoxygenated hemoglobin S (HbS), and resulting in small vessel obstruction by sickle-shaped erythrocytes. The understanding of SCD pathophysiology has greatly progressed over the last years, revealing multicellular cascades driven by inflammatory stimuli [2, 3]. SCD can now be considered a chronic inflammatory disease associated with increased levels of multiple cytokines during both vaso-occlusive crisis (VOC) and steady state [4–7]. The list of these pro-inflammatory cytokines, such as TNF-α, IL-1β and IL-6, has recently been extended to IFNγ and IL-17A (hereafter referred to as IL-17), which are classically produced not only by conventional Th1 and Th17 CD4^+^ T cells, but also by innate-like T cells [8–10].

Innate-like T (ILT) cells are unique unconventional lymphocytes sharing features of both innate and adaptive immune systems. They include invariant natural killer T (iNKT), mucosal-associated invariant T (MAIT) and gammadelta (γδ) T cells, which are characterized by a restricted T cell receptor (TCR) usage [11]. iNKT and MAIT cells express an antigen-specific semi-invariant TCR, TRAV10-TRAJ18 and TRAV1-2-TRAJ33, respectively [12]. γδ T cells are not a homogeneous population but the Vδ2^+^ subset is predominant in human peripheral blood [12, 13]. By contrast with conventional T cells, which recognize peptides, iNKT cells are activated by glycolipids presented by CD1d, MAIT cells are targeted by vitamin B metabolites presented by the MHC-related protein 1 (MR1) molecules, and γδ T cells are activated by a wide range of antigens without requiring MHC or MHC-related molecules [12–14]. These cells are able to produce large amounts of cytokines shortly after stimulation and play an important role in first-line defense against microbial infections [15–18]. However, ILT cells are also involved in a growing number of inflammatory diseases, as they can shift toward a pro-inflammatory state, with increased production of pathogenic cytokines, including IL-17 [19–24].

Recent studies have highlighted the possible implication of ILT cells in the inflammatory condition associated with SCD. Increased numbers of circulating iNKT cells with upregulated activation markers and increased IFNγ production during VOC have been reported in adult SCD patients versus healthy controls [25–27]. However, these results have not been confirmed in children and, to our knowledge, the possible implication of MAIT and γδ T cells in SCD pathophysiology has not been investigated so far. In the present study we address this issue by assessing the number and the cytokine-producing capacities of conventional as well as ILT lymphocytes, namely iNKT, MAIT and Vδ2 T cells, in the peripheral blood of children with SCD compared to age-matched controls of Afro-Caribbean origin.

## Methods

### Study design

Our monocentric prospective observational study was performed between 2017 and 2018 in a pediatric French university hospital SCD reference center. Patients of all SCD types, including HbSS, HbSC, HbS/β^0^ and HbS/β^+^, were eligible at an age of ≥ 1 year. Exclusion criteria were other diseases potentially affecting ILT cells (e.g., asthma, inflammatory or infectious diseases), treatments that could modify ILT cells (e.g., immunosuppressive therapies) and recent VOC (<1 month) for steady state patients or patients in a monthly exchange transfusion (MET) program. All patients were recruited: 1) during a routine visit, in steady state; 2) during a MET; or 3) during hospitalization for VOC. Controls were recruited among unaffected siblings (HbAA) of SCD patients. Blood samples were collected in ethylenediamine tetraacetic acid, at the end of the routine visit, immediately before transfusion for patients on a MET program and during hospitalization for patients undergoing VOC.

The medical files of all patients were analyzed, and several clinical and biological data were recorded (Table 1).

**Table 1:** Main clinical and biological characteristics of patients and controls.

	*Patients during VOC*	*Patients in steady state*	*Patients during MET program*	*Controls*	P*
*Number*	13	14	12	11	
*Age (years)*, *median [IQR]*	11.0 [9.0–13.7]	12.8 [10.8–14.7]	12.2 [8.9–15.9]	9.3 [4.9–15.3]	0.26
*Sex (female/male)*	5/8	7/7	3/9	6/5	0.18
*SCD type (SS/Sβ*^*0*^*/SC)*	10/2/1	13/1/0	12/0/0	-	-
*Hydroxyurea*	61.5% (n = 8)	78.6% (n = 11)	33.3% (n = 4)	-	-
*Number of VOC in the previous year*	1.8 +/- 1.4	1.1 +/- 1.9	0.2 +/- 0.6	-	-
*Number of ACS in the previous year*	0.2 +/- 0.4	0.1 +/- 0.4	0.2 +/- 0.6	-	-
*HbS (%)*	-	84.1 [77.4–90.6]	28.5 [24.5–34.4]	-	-
*HbF (%)*	-	4.6 [3.1–7.3]	1.6 [1.5–3.2]	-	-
*Hemoglobin (g/dL)*	9.0 [8.2–9.6]	7.8 [7.2–8.5]	10.2 [9.7–11.4]	12.7 [11.8–13.5]	**<0.0001**
*Reticulocyte count (G/L)*	269 [239–355]	262 [205–306]	511 [400–525]	50 [33–68]	**<0.0001**
*Leukocyte count (G/L)*	10.5 [8.8–12.4]	9.8 [8.3–11.3]	10.6 [8.9–13.3]	6.5 [4.9–8.0]	**<0.0001**
*Lymphocyte count (G/L)*	3.7 [2.6–4.4]	3.5 [2.8–4.3]	2.8 [2.5–3.2]	3.1 [2.6–3.3]	0.22
*LDH (U/L)*	-	478 [435–588]	-	240 [186–303]	**0.002**
*Free bilirubin (μmol/L)*	-	38.0 [16.0–61.0]	32.5 [17.3–40.8]	6.5 [4.8–8.5]	**<0.0001**
*CRP (mg/L)*	27.2 [12.1–58.5]	-	1.5 [1.0–2.0]	-	-

Data are expressed as median [interquartile range], mean ± SD or percentage.

*Comparison between all SCD patients and controls. Results with a P-value <0.05 are indicated in bold.

ACS: acute chest syndrome. CRP: C-reactive protein. HbF: fetal hemoglobin. HbS: hemoglobin S. LDH: lactate dehydrogenase. MET: monthly exchange transfusion. SCD: sickle cell disease. VOC: vaso-occlusive crisis.

Informed consent was obtained from the parents or legal guardians and assent was obtained from the child when appropriate. The study was approved by a Medical Ethics Committee (GR-Ex/CPP-DC2016-2618/CNIL-MR01).

### Flow cytometry

Freshly isolated peripheral blood mononuclear cells (PBMC) were isolated by using Ficoll-Paque density centrifugation (1.077 g/mL; PAA Laboratories GmbH). Cells were then washed in fluorescence activated cell sorting (FACS) buffer (2% (w/v) Bovine Serum Albumin, 0.1% NaN3 sodium azide in Phosphate Buffered Saline) then stained with fluorochrome-conjugated antibodies for 30 minutes at 4°C. Doublets were excluded using pulse-height and pulse-area and side scatter properties. Fixable viability dye was used to exclude dead cells (ThermoFischer Scientific). MAIT cells (CD3^+^TCRVα7.2^+^CD161^+^) were identified by the following monoclonal antibodies: anti-CD3-AlexaFluor700 (BD Biosciences), anti-Vα7.2-FITC (Biolegend) and anti-CD161-BV785 (eBiosciences). iNKT cells were identified by using CD1-PBS57-APC tetramers (NIH Tetramer Core Facility) associated with anti-CD3-AlexaFluor700 mAb, while Vδ2^+^ γδ T cell subset was identified by anti-Vδ2-PE and anti-CD3-AlexaFluor700 mAbs (BD Biosciences).

For intracellular cytokine analysis allowing the identification of IL-17, IFNγ, IL-4 and IL-13-producing T cells, freshly isolated PBMC were cultured during 5 hours with 25 ng/mL phorbol-12-myristate 13-acetate (PMA) and 10^−6^ M ionomycin, in the presence of 10 ng/mL Brefeldin A (Sigma-Aldrich), as previously described [28, 29]. Following surface staining, cells were washed and fixed with 4% paraformaldehyde and permeabilized with 0.5% saponin (Sigma-Aldrich) before further incubation for 45 minutes with anti-IL-17-BV605, anti-IFNγ-BV711, anti-IL-4-PE-Cy7 (Sony Biotechnology) and anti-IL-13-V450 (BD-Biosciences) [22].

Cells were identified by flow cytometry using a FACS Fortessa^™^ (BD-Biosciences) and analyzed with FlowJo software. S1 Fig shows a representative FACS analysis illustrating how the distinct T cell populations were gated for cytokine assays.

### Statistical analysis

Data are expressed as median [interquartile range (IQR)] or percentage. Differences between groups were assessed with the Mann-Whitney test. Correlation analyses between cytokine production by conventional T cells and innate-like T cells and several clinical and biological factors (Table 1) were made using Spearman’s rank correlation analysis. Statistical significance threshold was set at a P-value of 0.05. Data were analyzed using GraphPad Prism (GraphPad Software).

## Results

### Patients

Our study comprised a cohort of 39 children with SCD (14 in steady state, 12 on a MET program and 13 undergoing VOC) and 11 age-matched healthy controls (Table 1). Patients were homozygous HbSS (n = 35, 89.7%), HbS/ß^0^ (n = 3, 7.7%) and HbSC (n = 1, 2.6%). Their median [IQR] age was 12.4 [9.3–15.0] years. Median basal hemoglobin level was 7.5 [7.2–8.3] g/dL and 8.5 [7.7–9.0] g/dL for patients receiving hydroxyurea. Twenty-three patients (59.0%) were currently receiving hydroxyurea with a median dose of 20 [19.5–22.5] mg/kg/day and median treatment duration of 3.5 (1.2–7.3) years. Twelve patients (30.8%) were on a MET program (median duration of 4.6 [1.2–6.4] years) for a history of stroke (33.3%), abnormal transcranial Doppler velocities (50.0%) or frequent VOC and acute chest syndromes (ACS) despite treatment with hydroxyurea (16.7%). For patients in VOC, flow cytometry analyses were performed after a median of 2.0 [2.0–3.0] days of pain.

#### Conventional T cells

**Fig 1:**
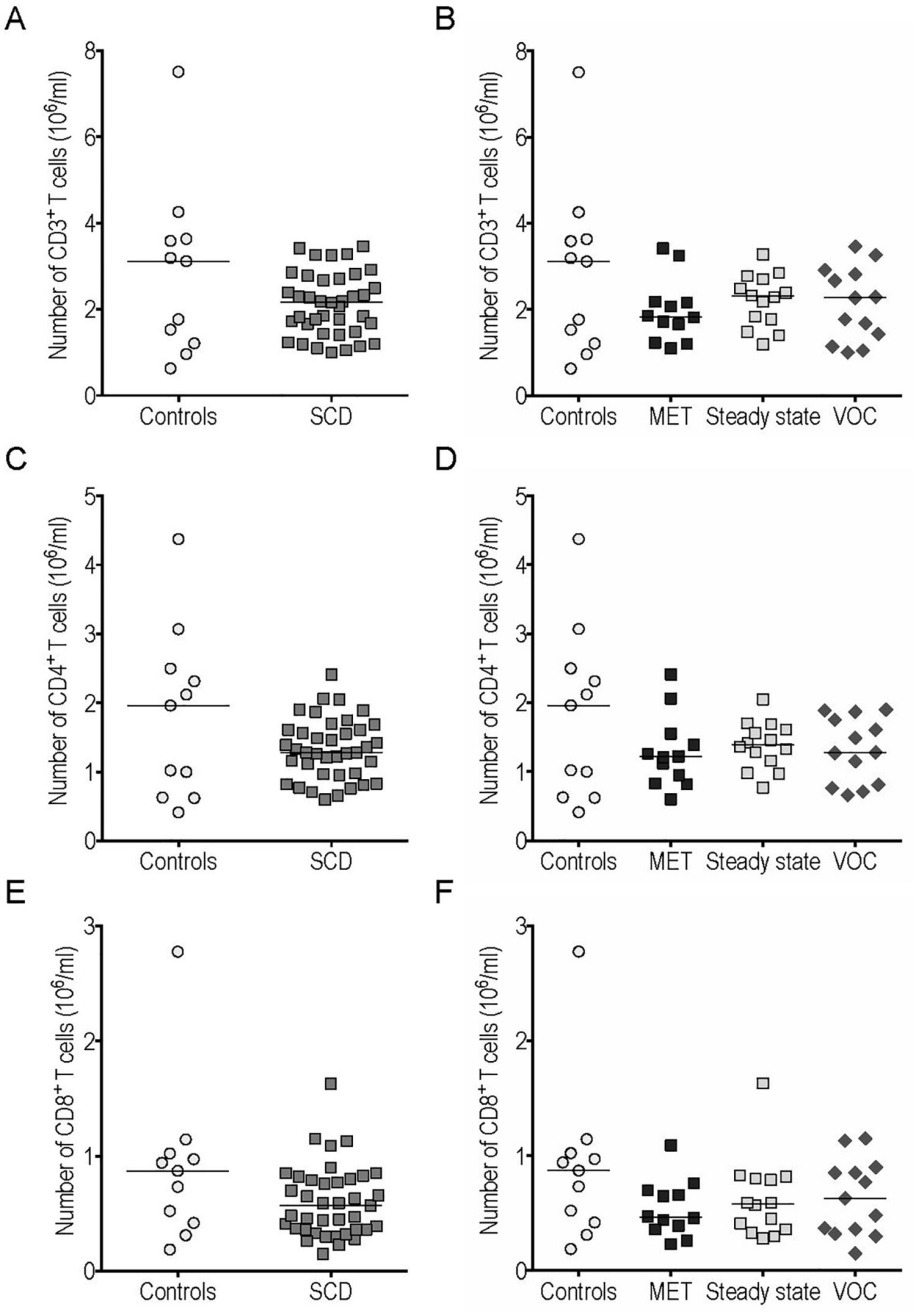
Conventional T cell counts in peripheral blood were similar in sickle cell disease (SCD) patients and controls. The CD3^+^ (A-B), CD4^+^ (C-D) and CD8^+^ (E-F) cell counts were compared between SCD patients and controls (A, C, E) and between different subgroups (B, D, F). MET: monthly exchange transfusion; VOC: vaso-occlusive crisis.

The number of CD3^+^ T cells among circulating lymphocytes was similar in SCD patients and controls (Fig 1A), as were conventional CD4^+^ and CD8^+^ T cell counts (Fig 1C and 1E). We found no significant difference between CD3^+^, CD4^+^ and CD8^+^ T cell counts in the 3 SCD subgroups (MET, steady state and VOC) (Fig 1B, 1D and 1F).

**Fig 2:**
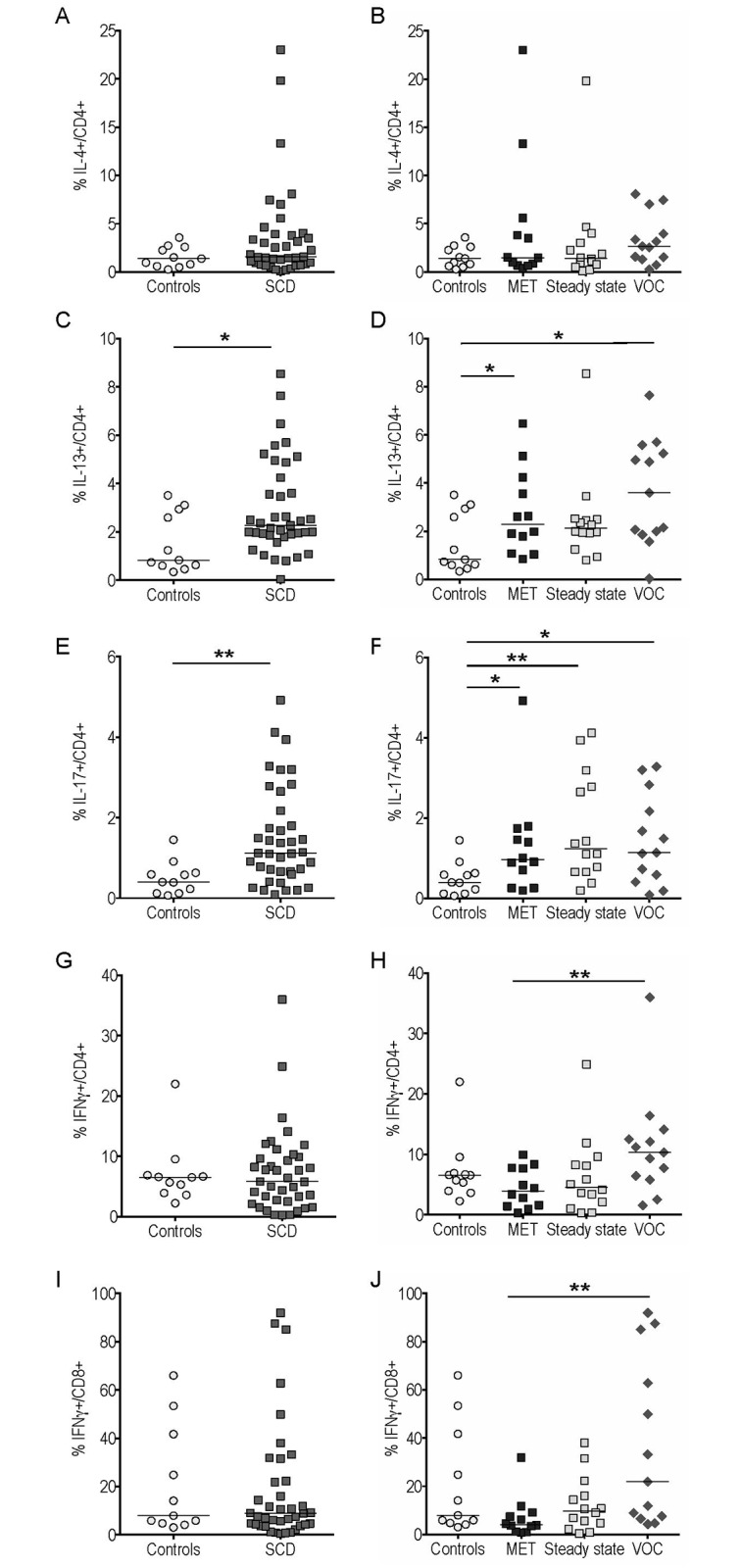
IL-13 and IL-17 production by conventional CD4^+^ T cells was increased in peripheral blood of sickle cell disease (SCD) patients compared to controls. The frequency of IL-4^+^ (A-B), IL-13^+^ (C-D), IL-17^+^ (E-F) cells among gated CD4^+^ cells and of IFNγ ^+^ (G-J) cells among gated CD4^+^ (G-H) and CD8^+^ (I-J) cells were compared between SCD patients and controls. *p<0.05; **p<0.01.

The frequency of IL-4^+^ cells among gated conventional CD4^+^ T cells was unchanged in SCD patients (whatever the subgroup), compared to controls (Fig 2A and 2B). By contrast, the proportion of CD4^+^ T cells expressing IL-13, another typical Th2 cytokine, was higher in SCD patients and remained significantly higher, when comparing MET and VOC groups with controls (Fig 2C and 2D).

A highly significant increase in the proportion of IL-17-producing CD4^+^ Th17 cells was observed in SCD patients (whatever the subgroup) versus controls (Fig 2E and 2F).

The frequency of IFNγ^+^ cells among gated CD4^+^ T cells was unchanged in SCD patients relative to controls (Fig 2G). However, the frequency of these IFNγ^+^ CD4^+^ Th1 cells was significantly enhanced in the VOC versus the MET group (Fig 2H). It is noteworthy that similar results were obtained regarding the frequency of IFNγ^+^ cells among gated CD8^+^ T cells (Fig 2I and 2J). Medians [IQR] and P-values <0.05 of the results presented in Figs 1 and 2 are summarized in S1 and S2 Tables.

#### Innate-like T cells

**Fig 3:**
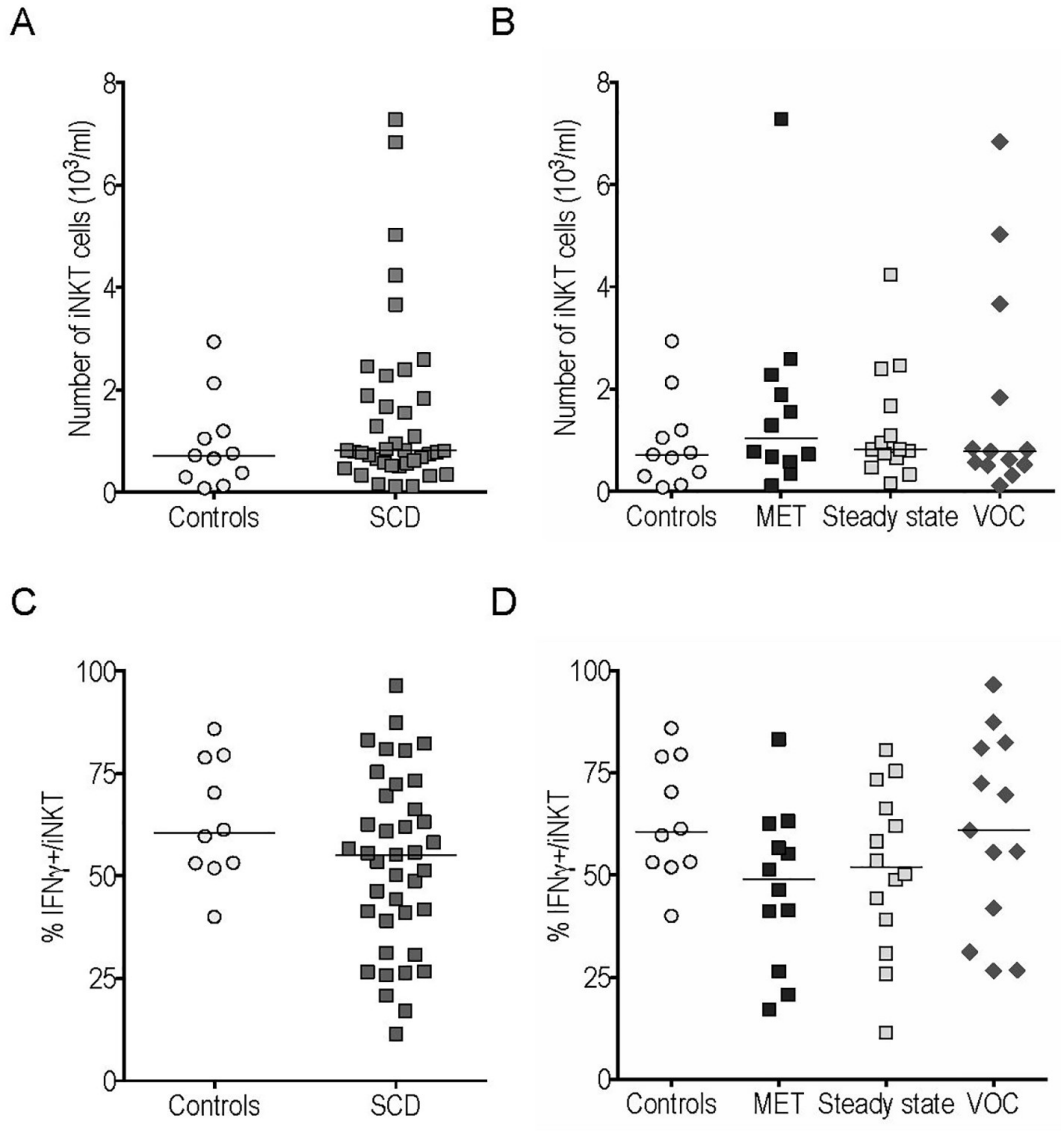
The number of iNKT cells and their intracellular cytokine production in peripheral blood were similar in sickle cell disease (SCD) patients and controls. iNKT cell counts (A-B) and frequency of IFNγ^+^ cells among gated iNKT cells (C-D) from SCD patients and controls.

We found no significant difference regarding iNKT cell counts and the frequency of IFNγ-producing iNKT cells between SCD patients, or any of the 3 SCD subgroups, and controls (Fig 3A–3D). However, a slight statistically non-significant trend toward increased IFNγ production by iNKT cells was observed during VOC compared with steady state and MET (p = 0.28 and p = 0.12).

**Fig 4:**
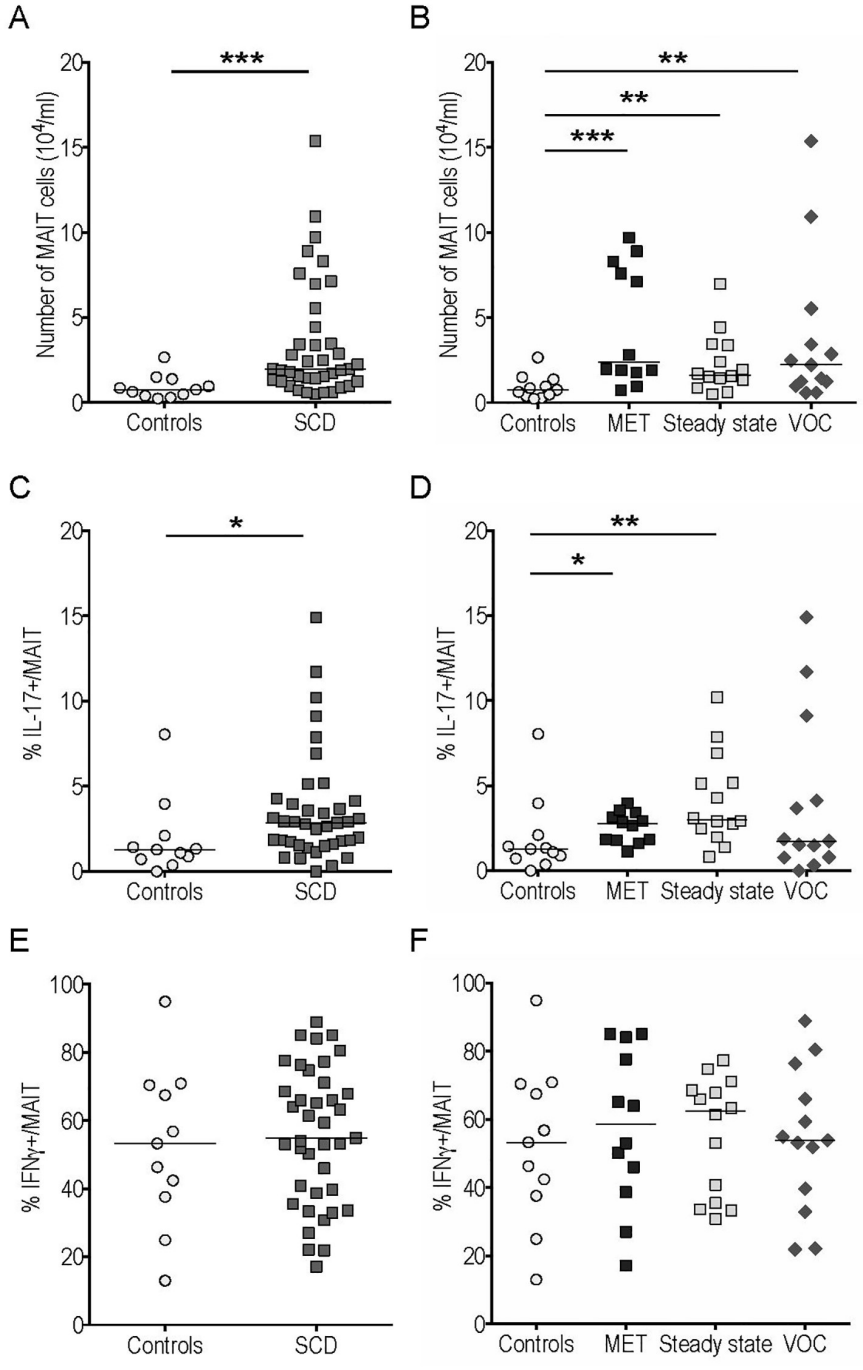
MAIT cell counts were increased with enhanced IL-17 production in peripheral blood of sickle cell disease (SCD) patients compared to controls. MAIT cell counts (A-B) and frequency of IL-17^+^ (C-D) and IFNγ^+^ (E-F) cells among gated MAIT cells from SCD patients and controls. *p<0.05; **p<0.01; ***p<0.001.

MAIT cell counts were significantly higher in SCD patients, whatever the subgroup considered (Fig 4A and 4B). The frequency of IL-17- but not of IFNγ-producing cells among this population was higher in SCD patients than in controls (Fig 4C and 4E). Similar results were obtained when comparing MET and steady state patients with controls, while no significant increase occurred during VOC (Fig 4D). The frequency of IFNγ^+^ cells among gated MAIT cells was similar in the three patient subgroups (Fig 4F).

**Fig 5:**
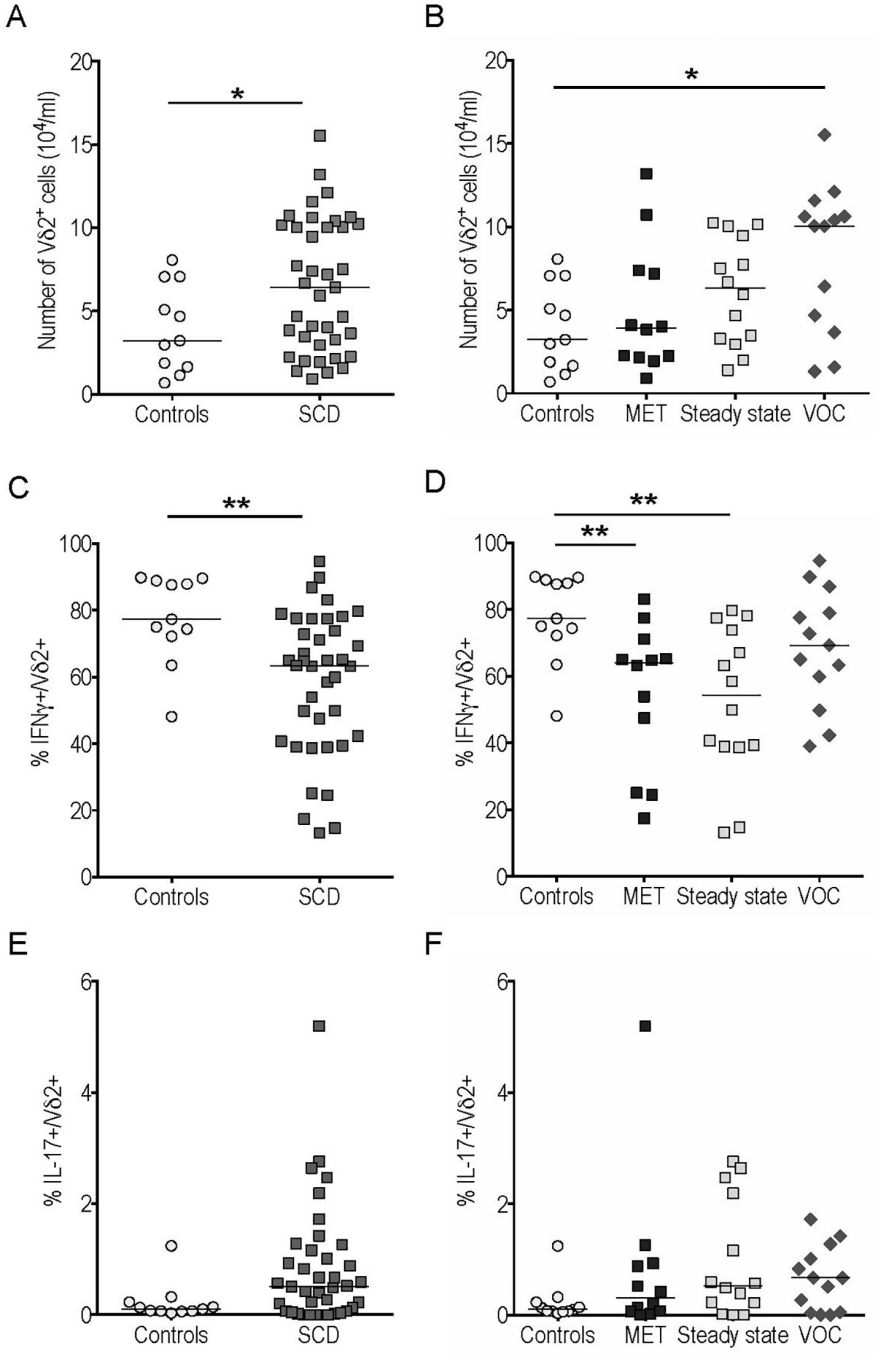
Vδ2 γδ T cell counts were increased with decreased IFNγ production in peripheral blood of sickle cell disease (SCD) patients compared to controls. Vδ2^+^ cell counts (A-B) and frequency of IFNγ^+^ (C-D) and IL-17^+^ (E-F) cells among gated Vδ2^+^ cells from SCD patients and controls. *p<0.05; **p<0.01.

The number of Vδ2 T cells, the major peripheral blood γδ T subset, was increased in SCD patients compared with controls, and further enhanced during VOC (Fig 5A and 5B). The frequency of IFNγ^+^ cells among this population was lower in SCD patients than in controls (Fig 5C), which was also the case for MET and steady state groups (Fig 5D). By contrast, a statistically non-significant trend toward increased IFNγ-producing Vδ2 T cells was observed during VOC compared to steady state (p = 0.07) (Fig 5D). IL-17-producing Vδ2 T cells also showed a tendency toward enhancement in SCD patients, without reaching statistical significance (p = 0.09) (Fig 5E). We found no significant difference between the patient subgroups in terms of IL-17 production by Vδ2 T cells (Fig 5F). Medians [IQR] and P-values <0.05 of the results presented in Figs 3–5 are summarized in S1 and S2 Tables.

#### Clinical and biological factors correlated with IFNγ and IL-17 production by conventional T cells and Innate-like T cells

The frequency of IFNγ^+^ cells among gated CD4^+^, CD8^+^ and Vδ2 T cells was positively correlated with the number of hospitalizations for VOC in the previous year (r = 0.45, p = 0.004; r = 0.51, p = 0.001; and r = 0.37, p = 0.021, respectively) and negatively with the time to last hospitalization for VOC (r = -0.38, p = 0.028; r = -0.45, p = 0.008; and r = -0.37, p = 0.029, respectively) (S2 Fig). The frequency of IFNγ^+^ cells among gated iNKT cells was positively correlated with patients’ age (r = 0.32, p = 0.048). The frequency of IL-17^+^ cells among gated Vδ2 T cells was positively correlated with HbS level (r = 0.47, p = 0.028). No other statistically significant correlation was found with any of the parameters listed in Table 1, including treatment with hydroxyurea.

## Discussion

In this study, we provide the first evidence for numerically and functionally distinct MAIT and Vδ2 ILT cells in children suffering from SCD relative to age-matched controls of Afro-Caribbean origin. We observed higher MAIT cell counts with an increased proportion of IL-17^+^ MAIT cells, and higher Vδ2 T cell counts, especially during VOC, with a decreased proportion of IFNγ^+^ Vδ2 T cells in off-crisis patients. These results are consistent with an involvement of innate-like T cells in SCD pathophysiology.

Higher levels of circulating iNKT cells have been previously reported in adult patients with SCD, together with increased production of IFNγ during VOC [25–27]. We could not reproduce these results in our pediatric population, besides a slight trend toward increased IFNγ production by iNKT cells during crisis. The small number of patients in our cohort, which reduces the statistical power, might explain this discrepancy. However, the positive correlation between the frequency of IFNγ-producing iNKT cells and patients’ age may also account for this difference.

Apart from iNKT cells, modifications of ILT populations had not yet been examined in SCD patients. We show here that both MAIT and γδ T cells may be involved in SCD pathophysiology, thus widening the spectrum of potential actors of innate immunity in SCD. Furthermore the increased Vδ2 T cell counts during VOC suggest a specific role for this ILT population during painful crises.

SCD can be viewed as an inflammatory disease leading to chronic inflammation, exacerbated during VOC [3, 30]. In addition to classical pro-inflammatory cytokines, such as TNF-α, IL-1β, IL-6, IL-8 and IFNγ, raised levels of IL-17 have been previously observed in the plasma of steady state SCD patients but the cells responsible for this activity were not clearly characterized [8–10]. We provide evidence for increased IL-17 production not only by CD4^+^ (Th17) but also by MAIT and possibly by γδ T cells in steady state SCD patients, as compared with controls. In these different T cell populations, IL-17 production did apparently not increase further during VOC, which is concordant with the data reported for IL-17 plasma levels [9, 10]. IL-17 is an essential pro-inflammatory T cell-derived cytokine which has been found to play a pivotal role in the defense against extracellular and fungal infections [31, 32]. However, this cytokine can also become deleterious, inasmuch as overwhelming production may lead to chronic inflammation and severe immunological conditions [32, 33]. IL-17 has pleiotropic effects on multiple target cells and promotes inflammation mainly by inducing several cytokines and chemokines, and enhancing neutrophil recruitment [34, 35]. During the last years, the crucial role of neutrophils in SCD has been largely described [3]. Moreover, IL-17 is a potent activator of the endothelium and can induce the expression of endothelial adhesion markers such as E-selectin, VCAM-1, and ICAM-1, which are known as major contributors to SCD pathophysiology [3, 5, 36]. Finally, IL-17 is involved in the pathogenesis of several autoimmune diseases, whose frequency seems to be increased in SCD [37, 38]. It has been previously suggested that IL-17 could represent a marker of SCD severity, as decreased IL-17 plasma levels have been observed in hydroxyurea-treated patients [9]. In addition, a trend toward decreased IL-17 levels has been documented in SCD patients with Benin/Benin haplotype, which gives rise to a less severe phenotype, compared to Bantu/Bantu haplotype [39]. In a similar line of evidence, we found a positive correlation between the frequency of IL-17-producing Vδ2 T cells and HbS level. Together with the fact that exchange transfusions reduce SCD severity by decreasing HbS level, these results support an association between IL-17 and SCD severity. Furthermore, it has been shown that IL-17 is a potent stimulant of lung microvascular endothelial cells to produce chemoattractants such as CXCL8, selectively driving neutrophil chemotaxis [36]. IL-17 may therefore play a role in the pathophysiology of acute chest syndrome (ACS). None of the patients included in our study suffered from ACS, but it might be relevant for future studies to analyze IL-17 production by Th17, MAIT and γδ T cells in SCD patients undergoing ACS.

Enhanced IL-4 secretion during VOC has been previously reported, suggesting a possible shift of the CD4^+^ T cell response towards a Th2 phenotype [40]. We found no significant difference regarding IL-4 production by CD4^+^ T cells, neither between children with SCD and controls, nor between VOC and other SCD subgroups. However, IL-13^+^ cells among gated CD4^+^ T cells were more frequent in patients than in controls, suggesting that the CD4^+^ T cell response is skewed toward Th2 and Th17 phenotypes with increased IL-13 and IL-17 production.

Higher IFNγ plasma levels have been observed in steady state SCD patients compared to controls, with a slight rise during VOC [8, 41]. Here, we report increased IFNγ production by CD4^+^, CD8^+^ and possibly by iNKT and Vδ2 T cells in patients undergoing VOC compared to off-crisis patients. Furthermore, we found that IFNγ production by CD4^+^, CD8^+^ and Vδ2 T cells was positively correlated with the frequency of VOC and negatively with the time to last hospitalization for VOC. These results suggest that IFNγ may be involved in VOC pathophysiology, whereas IL-17 essentially contributes to the chronic pro-inflammatory state of SCD. On the other hand, the reduced production of IFNγ by Vδ2 T cells in off-crisis SCD patients versus controls suggests chronic down-modulation of these cells, similarly to what has been described in other inflammatory disorders, such as Behcet’s disease [42].

It has been reported that hydroxyurea could have a potential modulatory impact on several immune cells but in our study, no correlation was found between hydroxyurea and any of the analyzed cell subsets [43].

Further studies are required for deciphering the mechanisms leading to the observed alterations of MAIT and γδ T cells in SCD patients. Microbial infections are well-known activators of ILT cells but all SCD patients included in our study were receiving antibiotic prophylaxis and no blood sample was collected during an infection. By analogy with other innate immune cells, a role for chronic hemolysis and inflammation may be suspected [3].

There are some limitations in our study, such as the small number of patients in each group, which may reduce statistical power. Nevertheless, several statistically significant differences between the patient groups could be identified. We are aware that the use of antigens presented by antigen-presenting cells (APC) would be more physiologically relevant than stimulation with PMA and ionomycin to detect the ability of peripheral blood T cells to secrete IL-17, even though these mitogens are currently used by other research groups [44, 45]. Unfortunately, antigen and APC stimulation requires a larger volume of blood than what we were authorized to collect. In addition, antigen stimulation induces T cell receptor (TCR) down-regulation, which may compromise the identification of MAIT cells.

In conclusion, this is the first demonstration in patients with SCD of increased MAIT cell counts with enhanced IL-17 production, increased Vδ2 T cell counts with decreased IFNγ production, and enhanced IL-13 and IL-17 production by CD4^+^ T cells, as compared to controls. It opens new perspectives for the study of innate-like T cells and Th17 cells, as likely important actors of SCD pathophysiology. Further studies are required to assess if these parameters could help predicting the severity of disease outcome. Since IL-17 inhibitors have shown promising results in treating other inflammatory diseases, a similar approach may be relevant for patients affected with SCD.

## Supporting information

S1 Fig Flow cytometric gating strategy. Flow cytometric gating strategy used to identify iNKT (CD3^+^ CD1d-PBS57 tetramer^+^), Vδ2 γδ T cells (CD3^+^ CD1d-PBS57 tetramer^-^ Vδ2^+^), MAIT (CD3^+^ CD1d-PBS57 tetramer^-^ Vδ2^-^ CD161^+^ TCRVα7.2^+^), CD4^+^ (CD3^+^ CD1d-PBS57 tetramer^-^ Vδ2^-^ CD161^-^ TCRVα7.2^-^ CD8^-^ CD4^+^) and CD8^+^ (CD3^+^ CD1d-PBS57 tetramer^-^ Vδ2^-^ CD161^-^ TCRVα7.2^-^ CD8^+^ CD4^-^) cells from peripheral blood and their ability to produce IL-4, IL-13, IL-17 or IFNγ.
(TIFF)

S2 Fig Correlation between the frequency of IFNγ ^+^ cells among gated CD4^+^ (A), CD8^+^ (B) and Vδ2^+^ (C) cells and the time to last hospitalization for vaso-occlusive crisis (VOC). (TIF)

S1 Table Number and cytokine production of conventional and innate-like T cells. (DOCX)

S2 Table P-values for statistically significant comparisons between patient groups. (DOCX)
