# Supplementary figures and images for: Innate-like T cells in children with sickle cell disease
Source: PLoS One. 2019 Jun 28;14(6):e0219047. doi: 10.1371/journal.pone.0219047 (PMC6599217; doi:10.1371/journal.pone.0219047)

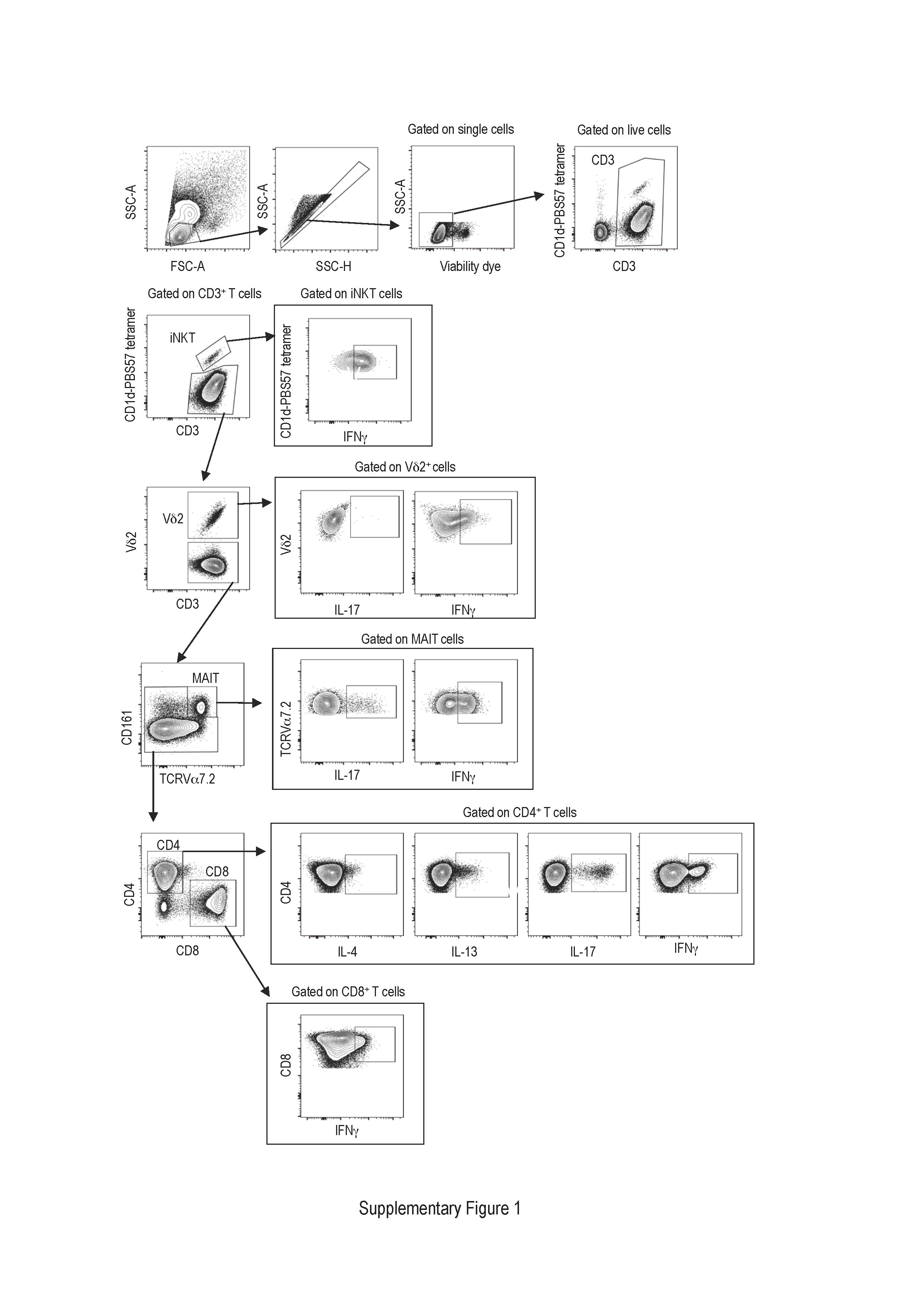

Supplement: S1 Fig — Flow cytometric gating strategy used to identify iNKT (CD3+ CD1d-PBS57 tetramer+), Vδ2 γδ T cells (CD3+ CD1d-PBS57 tetramer- Vδ2+), MAIT (CD3+ CD1d-PBS57 tetramer- Vδ2- CD161+ TCRVα7.2+), CD4+ (CD3+ CD1d-PBS57 tetramer- Vδ2- CD161- TCRVα7.2- CD8- CD4+) and CD8+ (CD3+ CD1d-PBS57 tetramer- Vδ2- CD161- TCRVα7.2- CD8+ CD4-) cells from peripheral blood and their ability to produce IL-4, IL-13, IL-17 or IFNγ. (TIFF) [file pone.0219047.s001.tiff]

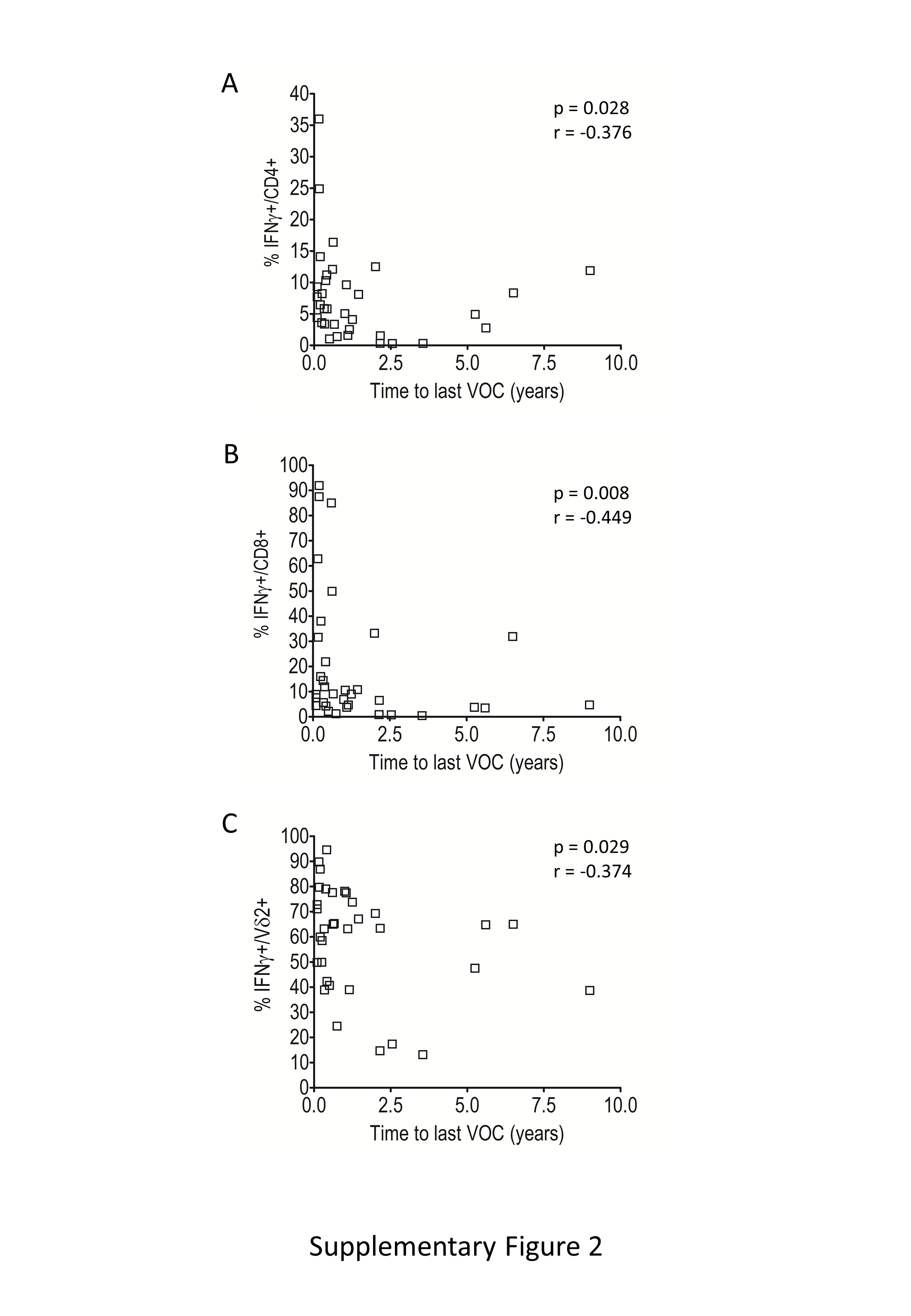

Supplement: S2 Fig — (TIF) [file pone.0219047.s002.tif]
