# Supplementary material for: Innate-like T cells in children with sickle cell disease
Source: PLoS One. 2019 Jun 28;14(6):e0219047. doi: 10.1371/journal.pone.0219047 (PMC6599217; doi:10.1371/journal.pone.0219047)
Supplement: S1 Table — (DOCX) [file pone.0219047.s003.docx]

**S1 Table. Number and cytokine production of conventional and innate-like T cells**

|  | Controls | SCD | MET | Steady state | VOC |
| --- | --- | --- | --- | --- | --- |
| CD3^+^ (10^6^/mL) | 3.1 [0.6-7.5] | 2.2 [1-3.5] | 1.8 [1.1-3.4] | 2.3 [1.2-3.3] | 2.3 [1-3.5] |
| CD4^+^ (10^6^/mL) | 1.9 [0.4-4.4] | 1.3 [0.6-2.4] | 1.2 [0.6-2.4] | 1.4 [0.8-2.0] | 1.3 [0.7-1.9] |
| CD8^+^ (10^6^/mL) | 0.9 [0.2-2.8] | 0.6 [0.1-1.6] | 0.5 [0.2-1.1] | 0.6 [0.3-1.6] | 0.6 [0.1-1.1] |
| IL-4^+^/CD4^+^ (%) | 1.4 [0.3-3.6] | 1.6 [0.1-23] | 1.5 [0.4-23] | 1.4 [0.1-19.8] | 2.7 [0.3-8] |
| IL-13^+^/CD4^+^ (%) | **0.8 [0.3-3.5]** | **2.3 [0.04-8.5]** | **2.3 [0.8-6.5]** | 2.1 [0.8-8.5] | **3.6 [0.04-7.6]** |
| IL-17^+^/CD4^+^ (%) | **0.4 [0.07-1.4]** | **1.1 [0.1-4.9]** | **0.9 [0.2-4.9]** | **1.2 [0.2-4.1]** | **1.1 [0.1-3.3]** |
| IFNγ^+^/CD4^+^ (%) | 6.5 [2.2-22] | 5.8 [0.3-36] | **3.9 [0.3-9.9]** | 4.6 [0.3-24.9] | **10.3 [1.5-36]** |
| IFNγ^+^/CD8^+^ (%) | 7.8 [3.0-66] | 8.8 [0.4-91.9] | **4.1 [0.8-31.9]** | 9.8 [0.4-38] | **21.9 [4.3-91.9]** |
| iNKT (10^3^/mL) | 0.72 [0.08-2.94] | 0.82 [0.12-7.28] | 1.04 [0.12-7.28] | 0.82 [0.16-4.24] | 0.79 [0.12-6.84] |
| IFNγ^+^/ iNKT (%) | 60.5 [40-85.9] | 55.2 [11.5-96.5] | 48.8 [17-83.2] | 51.8 [11.5-80.6] | 60.9 [26.6-95.5] |
| MAIT (10^4^/mL) | **0.75 [0.23-2.65]** | **1.95 [0.49-15.4]** | **2.40 [0.72-9.70]** | **1.63 [0.49-6.98]** | **2.22 [0.57-15.4]** |
| IL-17^+^/MAIT (%) | **1.3 [0.01-8.04]** | **2.9 [0.01-14.9]** | **2.7 [1.1-3.9]** | **3.0 [0.8-10.2]** | 1.7 [0.01-14.9] |
| IFNγ^+^/ MAIT (%) | 53.3 [13-94.9] | 54.9 [17.1-88.9] | 58.5 [17.1-85.1] | 62.3 [30.8-77.3] | 53.9 [21.9-88.9] |
| Vδ2 (10^4^/mL) | **3.23 [0.69-8.01]** | **6.43 [0.92-15.5]** | 3.93 [0.92-13.2] | 6.31 [1.39-10.2] | **10.0 [1.32-15.5]** |
| IFNγ^+^/ Vδ2 (%) | **77.3 [48.1-89.8]** | **63.4 [13.2-94.6]** | **64 [17.4-83.1]** | **54.2 [13.2-79.7]** | 69.3 [39.1-94.6] |
| IL-17^+^/ Vδ2 (%) | 0.1 [0.03-1.24] | 0.51 [0.01-5.2] | 0.31 [0.01-5.2] | 0.53 [0.01-2.76] | 0.67 [0.01-1.7] |

Data are expressed as median [interquartile range]. Comparisons with a P-value <0.05 are indicated in bold.
