# Supplementary material for: Innate-like T cells in children with sickle cell disease
Source: PLoS One. 2019 Jun 28;14(6):e0219047. doi: 10.1371/journal.pone.0219047 (PMC6599217; doi:10.1371/journal.pone.0219047)
Supplement: S2 Table — (DOCX) [file pone.0219047.s004.docx]

**S2 Table. P-values for statistically significant comparisons between patient groups**

| P-value | SCD vs Controls | MET vs Controls | Steady state vs Controls | VOC vs Controls | VOC vs MET |
| --- | --- | --- | --- | --- | --- |
| IL-13^+^/CD4^+^ (%) | 0.02 | 0.044 | - | 0.018 | - |
| IL-17^+^/CD4^+^ (%) | 0.001 | 0.02 | 0.003 | 0.02 | - |
| IFNγ^+^/CD4^+^ (%) | - | - | - | - | 0.007 |
| IFNγ^+^/CD8^+^ (%) | - | - | - | - | 0.002 |
| MAIT (10^4^/mL) | 0.0003 | 0.0005 | 0.006 | 0.009 | - |
| IL-17^+^/MAIT (%) | 0.02 | 0.04 | 0.01 | - |  |
| Vδ2 (10^4^/mL) | 0.03 | - | - | 0.018 | - |
| IFNγ^+^/ Vδ2 (%) | 0.005 | 0.005 | 0.004 | - | - |
